# Effects of Growth Stage and Physiological State on Gut Health, Antioxidant Capacity, and Muscle Nutritional Composition in Greenhouse-Cultured *Pelodiscus sinensis*

**DOI:** 10.3390/antiox15091100

**Published:** 2026-08-31

**Authors:** Qingman Yang, Yuhong Liao, Huihui Quan, Qing Qiu, Huahua Mao, Dachun Gong, Xuan Tu, Zirui Wang, Hao Fu, Xionge Pi

**Affiliations:** 1 Shaoxing Fisheries Technical Extension Center, Shaoxing 312000, China; 13205851638@163.com (Q.Y.);; 2 Zhejiang Provincial Key Laboratory of Agricultural Microbiomics, Institute of Plant Protection and Microbiology, Zhejiang Academy of Agricultural Sciences, Hangzhou 310021, China; 19379171107@163.com; 3 College of Biology and Pharmacy, China Three Gorges University, Yichang 443002, China; 4 College of Animal Science and Technology, Jiangxi Agricultural University, Nanchang 330045, China; wangzirui@jxau.edu.cn

**Keywords:** *Pelodiscus sinensis*, gut microbiota, antioxidant capacity, physiological stage, healthy state, sustainable cultivation

## Abstract

The production performance, muscle quality, and gut microbiota of aquatic animals are closely associated with host growth stage and disease status. This study therefore compared the gut health, antioxidant capacity, and muscle nutritional composition of greenhouse-cultured *Pelodiscus sinensis* covering two growth stages (juvenile and adult) and two physiological states (healthy and diseased). The results showed that healthy turtles possessed significantly longer and denser intestinal villi compared with diseased turtles (*p* < 0.01), and adult turtles maintained higher activities of glutathione peroxidase (GSH-Px), superoxide dismutase (SOD), and lysozyme (LZM) than juveniles regardless of health condition. Digestive enzyme profiles varied with age and health. Compared with adult turtles, juveniles exhibited significantly higher trypsin activity in both healthy and diseased groups (*p* < 0.001). Conversely, juveniles displayed significantly lower lipase activity than adults in the diseased group (*p* = 0.008). For muscle nutritional composition, adults contained higher crude protein (CP), essential amino acids (EAAs), and umami amino acids (UAAs) than juveniles, while healthy turtles had significantly better muscle quality indices than diseased individuals (*p* < 0.05). Meanwhile, gut microbial composition differed significantly between growth stages and health states (*p* = 0.001). The relative abundance of *unclassified_f_Lachnospiraceae* and *Clostridium_sensu_stricto_2* was positively correlated with GSH-Px and muscle nutritional composition indicators (CP, EAAs, and UAAs), whereas it was negatively correlated with liver alkaline phosphatase (ALP), acid phosphatase (ACP), and intestinal enzyme activities. Data also showed that *unclassified_o_Bacteroidales* and *unclassified_f_Lachnospiraceae* may affect muscle nutritional composition and non-specific immunity by regulating the microbial functional pathways of amino acid metabolism, the digestive system, and non-specific immunity system. Collectively, these observations offer insights into variations in physiological traits of greenhouse-cultured *P. sinensis*. These correlational findings suggest that gut microbiota are closely associated with muscle nutrition and antioxidant status, providing valuable insights for sustainable soft-shelled turtle aquaculture.

## 1. Introduction

The *P. sinensis* (Chinese soft-shelled turtle, hereinafter referred to as turtle) is an aquatic economic species that possesses substantial economic value within China’s freshwater aquaculture industry. This is attributed to the optimal nutritional composition and high nutritional content of its meat [1]. China produces over 95% of the world’s turtle production, with an annual output exceeding 400,000 tons. To satisfy market demand, intensive farming models, such as greenhouse farming and pond farming, have gained considerable popularity in the southeastern part of mainland China. These models aim to augment yield and shorten the growth cycle through regulating the aquaculture environment and facility conditions [2]. Nonetheless, turtles are vulnerable to infection by diverse pathogens during the farming process [3], including bacteria [4], viruses [5], and parasites [6]. Particularly under intensive farming conditions, they are prone to attacks by multiple pathogens, such as iridovirus [5], hemorrhagic syndrome virus [7], *Aeromonas hydrophila* [8], and *Bacillus* spp. [9]. These pathogens can induce diseases like fulminant hemorrhagic disease, white abdominal disease, and mumps, leading to significant aquaculture losses [10]. Currently, the prevention and treatment methods for these diseases remain undefined. Owing to the frequent occurrence of disease in turtles, farmers have long relied on antibiotics for disease prevention and treatment [11]. This not only results in elevated aquaculture costs, but also gives rise to food safety issues, such as intestinal flora imbalance, increased drug resistance of pathogenic bacteria, and excessive drug residues in meat products due to over-medication [12], severely impeding the sustainable development of the industry.

In an intensive greenhouse farming model, white abdominal disease (WAD) exhibits a high incidence rate, resulting in elevated mortality, reduced growth performance, and impaired meat quality, all of which collectively compromise turtle productivity and economic returns [13,14]. As a highly contagious and multifactorial syndrome, the pathogenic mechanisms of WAD remain poorly understood, and the body of theoretical evidence supporting related basic research remains limited. Accumulating evidence suggests associations with bacterial, viral, and nutritional factors [10,15]. Moreover, studies by Nash et al. have noted that this disease causes inflammatory responses and immune system dysfunctions in turtles [15]. The infected or diseased state is associated with increased oxidative stress, which further degrades the muscle nutritional composition [16]. Investigations have revealed that antioxidant capacity and non-specific immunity serve as crucial defense mechanisms against pathogenic incursions [17]. The antioxidant and immune capabilities of turtles display notable variations across different growth phases and physiological states [18]. For example, Zhao et al. verified that the immune response of Chinese soft-shelled turtles suffering from WAD is significantly compromised [19].

Recent research endeavors have demonstrated that the gut microbiota assume a pivotal role in host digestion, nutrient assimilation, and immune reactions [18]. Moreover, it exhibits a close correlation with the nutritional composition of the muscle tissue [20]. Significantly, notable disparities in gut microbiota are discernible among animals of distinct breeds, growth phases, and dietary patterns [21,22]. Concurrently, the gut microbiota of aquatic animals is also subject to the influence of the aquaculture water milieu [23]. At present, the approach of enhancing animal immunity through the regulation of gut microbiota to augment animal production performance and improve muscle quality has emerged as a prominent research focus [24]. In this context, considerable headway has been achieved in modulating the growth performance, immune-related enzyme activities, and gut microbiota of turtles via diverse dietary constituents [25]. However, comparisons between healthy and diseased individuals under specific farming models are predominantly confined to single indicators (such as immune indicators or microbiota structure) [26]. This limitation has prevented the integrated analysis of intestinal microbiota, antioxidant activity, intestinal barrier integrity, and muscle nutritional components, thereby impeding a comprehensive understanding of the interaction mechanisms among physiological systems.

## 2. Materials and Methods

### 2.1. Sample Collection

Sampled male turtles were randomly chosen from a greenhouse at the Jindadi Turtle and Soft-Shelled Turtle Farming Company in Shaoxing City, Zhejiang Province, on 15 August 2024 (N: 29° 55′ 52″, E: 120° 13′ 43″). Healthy turtles exhibited normal feeding behavior and showed no visible signs of disease. The diseased turtles suffered from WAD, showing reduced feeding activity, with prominent red lesions on their abdomens. Upon dissection, the intestines of these infected individuals displayed signs of hemorrhaging. Pathogen testing confirmed infection with *Aeromonas hydrophila*. The aquaculture parameters were set as follows: water temperature was maintained at 30.0 ± 0.5 °C; the photoperiod was a 12 h light/12 h dark cycle; and the experimental diet was provided twice daily at 08:00 and 20:00 by feed products procured from Haihuang Technology Co., Ltd. (Hangzhou, China), accounting for 3% of the turtle’s body weight per day (diet and nutritional composition detailed in Table S1). This animal study was approved by the Institution of Animal Care and Use Committee (ACUC) of Zhejiang Academy of Agricultural Sciences (No. 2023ZAASLA58) on 20 June 2023.

The study was conducted in 20 greenhouse rearing pools under strictly controlled environmental conditions. A total of ten juvenile turtles (average weight: 100 ± 5.0 g, two months old) were randomly collected from ten aforementioned independent greenhouses, including five healthy individuals and five diseased individuals, and divided into healthy juvenile turtle (HJI, *n* = 5) and diseased juvenile turtle (DJI, *n* = 5) groups. Each turtle was sampled from a greenhouse pool as an independent experimental unit. Additionally, ten adult turtles (average weight: 600 ± 20 g, eight months old) were also collected from ten independent greenhouses with a consistent aquaculture environment, including five healthy and five diseased individuals, referred to as healthy adult turtles (HAI, *n* = 5) and diseased adult turtles (DAI, *n* = 5). The flowchart of the experimental design is shown in Figure S1.

All turtles were transported to the laboratory, following a 24 h acclimation period in standard housing conditions (water temperature 28 °C, basking site 33 °C, 12L:12D photoperiod). Euthanasia was performed by anesthetizing the experimental animals with tricaine methanesulfonate (juvenile turtles: 300 mg/L; adult turtles: 500 mg/L). Death was confirmed by double spinal cord transection upon complete cessation of reflexes. The colon and small intestine tract were aseptically excised and cleared of adipose tissue, then intestinal contents were collected into pre-chilled 2 mL sterile tubes. Colon, liver, and hind leg muscle tissue were also obtained. Samples were flash-frozen in liquid nitrogen and stored at −80 °C until analysis.

### 2.2. Histological Observation of Colonic Tissue

After washing the colonic tissue of the turtles with sterile physiological saline, the samples were rapidly fixed in 4% paraformaldehyde solution. Then, the tissues were embedded in paraffin after 24 h of fixation. Longitudinal sections were cut to a thickness of 4 µm and dehydrated using an automatic dehydrator (Dakewe Biotech Co., Ltd., Shenzhen, China). The sections were stained with hematoxylin and eosin (H&E). Tissue morphology was observed using a digital pathology scanner (Kfbio, Ningbo, China) with an optical microscope (Nikon, Tokyo, Japan). Histological parameters were measured using image analysis application software Sigma Scan Pro5 (Sigma-Aldrich Corporation, St. Louis, MO, USA), as described by an earlier report [27].

### 2.3. Composition of Amino Acids and Other Nutritional Components in Muscle

Fifty grams of muscle tissue were isolated from the hind legs, frozen in liquid nitrogen, and subsequently ground into a fine powder with a mortar. The samples were then hydrolyzed in 6 mol/L hydrochloric acid (HCl) for 24 h, and then the HCl was removed using a rotary evaporator (Heidolph Laborota 4000, Heidolph Instruments GmbH & Co. KG, Schwabach, Germany). The contents of amino acids were quantified using an automatic amino acid analyzer (Hitachi L8900, Hitachi, Ltd., Tokyo, Japan). Crude protein content was determined using the Kjeldahl method, while crude fat content was assessed via the Soxhlet extraction method by utilizing petroleum ether as the solvent [28].

### 2.4. Determination of Enzyme Activity in Liver and Intestinal Tissues

Liver tissue and small intestine samples were thawed on ice, and liver samples were homogenized for further measurement. Commercially available assay kits (Nanjing Jiancheng Bioengineering Institute, Nanjing, China) were used to measure indicators of antioxidant activity, non-specific immunity, and intestinal digestive enzymes following manufacturer protocol. Five liver antioxidant enzymes were assessed using a spectrophotometer (Shimadzu, Kyoto, Japan) and colorimetric assays: total antioxidant capacity (T-AOC) (A015-2-1), catalase (CAT) activity (A007-1-1), glutathione peroxidase (GSH-Px) activity (A005-1-1), superoxide dismutase (SOD) activity (A001-3-2), and malondialdehyde (MDA) concentration (A015-2-1). Additionally, three hepatic innate immune enzymes were evaluated: lysozyme (LZM) activity (A050-1-1) was determined by turbidimetric assay, while alkaline phosphatase (ALP) (A059-1-1) and acid phosphatase (ACP) (A060-1-1) activities were measured using colorimetric assays on a spectrophotometer. In the small intestine, three digestive enzymes were analyzed: amylase (AMS) activity was measured using a colorimetric assay with a spectrophotometer (A082-1-1), while trypsin activity (A080-1-1) and lipase (LPS) activity (A054-1-1) were quantified using microplate readers. All assay kits were provided by Nanjing Jiancheng Bioengineering Institute, and absorbance was recorded using a UV-2600 spectrophotometer (Shimadzu, Kyoto, Japan) or a Multiskan FC microplate reader (Thermo Fisher Scientific, Waltham, MA, USA).

### 2.5. 16S rRNA Gene Sequencing Analysis of Gut Microbiota

Colonic contents from all Chinese soft-shelled turtles were processed according to experimental protocols to extract DNA using the DNeasy PowerSoil Pro Kit (Qiagen, Gaithersburg, MD, USA). The concentration and quality of the extracted DNA were assessed using a 1% agarose gel and quantified with a NanoDrop 2000 (Thermo Fisher Scientific, MA, USA). The bacterial 16S rRNA gene with V3–V4 hypervariable regions was amplified using the following primers: 338F-ACTCCTACGGGAGGCAGCAG and 806R-GGACTACHVGGGTWTCTAAT. The PCR reaction mixture included 10 μL of 5× fast reaction buffer, 1.5 μL of 2.5 mM dNTPs, 1.5 μL of each primer (10 μM), 0.2 μL of fast DNA polymerase, 50 ng of template DNA, and ddH_2_O at a final volume of 50 μL. The PCR amplification conditions included an initial denaturation at 95 °C for 5 min, followed by 30 cycles of denaturation at 95 °C for 1 min, annealing at 60 °C for 1 min, and extension at 72 °C for 1 min, concluding with a final extension at 72 °C for 7 min, and ending at 4 °C. Following purification using the PureLink Combo Kit, the PCR products were subjected to paired-end sequencing on the Illumina MiSeq PE300 platform (Illumina, San Diego, CA, USA). The 16S rRNA gene sequencing data were submitted to the NCBI database (accession number PRJNA1188359).

Raw sequencing reads were decompressed and quality filtered by fastp (v0.20.0). The reads were subsequently merged into tags using FLASH (v1.2.7) [29]. Chimeric sequences were filtered out using Vsearch software (v2.3.4). After denoising with DADA2, feature tables and sequences were generated. Each representative sequence of an amplicon sequence variant (ASV) table was annotated by a naive Bayesian classifier based on the SILVA database (v138). Feature abundance was normalized according to relative abundance in each sample, and the ASV table was rarefied based on the minimum sequencing count across all samples. Raw sequencing counts were normalized using centered log-ratio (CLR) transformation to mitigate compositional bias. Alpha diversity indices (Shannon index) were calculated using QIIME2 to evaluate the complexity of the microbial communities [30]. Beta diversity analysis was performed using principal coordinate analysis (PCoA) to investigate differences within and between groups. Furthermore, the relative abundance of bacteria exceeding 0.1% in each sample was analyzed using Python (v2.7). The R software package (v3.3.1) was used to assess the significance of species that exhibited significant differences across multiple groups.

### 2.6. Statistical Analysis

A total of 1,259,118 high-quality bacterial 16S rRNA gene sequences were obtained from intestinal samples, and 4914 operational taxonomic units (ASVs) were identified across all samples, providing a robust foundation for downstream microbial community analysis. The α-diversity indices of the microbiota, histomorphological parameters of the intestine, enzyme activity, and muscle nutritional content were expressed as the mean ± standard deviation (SD) in each group (*n* = 5). The distribution characteristics of all data were tested using the Shapiro–Wilk test. For multiple group comparisons, homogeneity of variance was verified by Bartlett’s test, then the Scheirer–Ray–Hare test was conducted for data with non-normal distributions, while two-way ANOVA combined with F-tests was applied for normally distributed data. Data analysis and graphical representations were conducted using GraphPad Prism 8.0.1. Additionally, Spearman’s rank correlation analysis was employed to evaluate the relationships among enzyme activity, muscle nutritional composition, and gut microbiota. The range for Spearman’s correlation coefficient (r) was set at −0.5 to 1.0, and statistical significance was set at *p* < 0.05. The correlation heatmap was generated using OmicStudio tools at https://www.omicstudio.cn/tool (accessed on 6 August 2025).

## 3. Results

### 3.1. Comparison of Intestinal Morphological and Digestive Enzyme Activity

**Figure 1:**
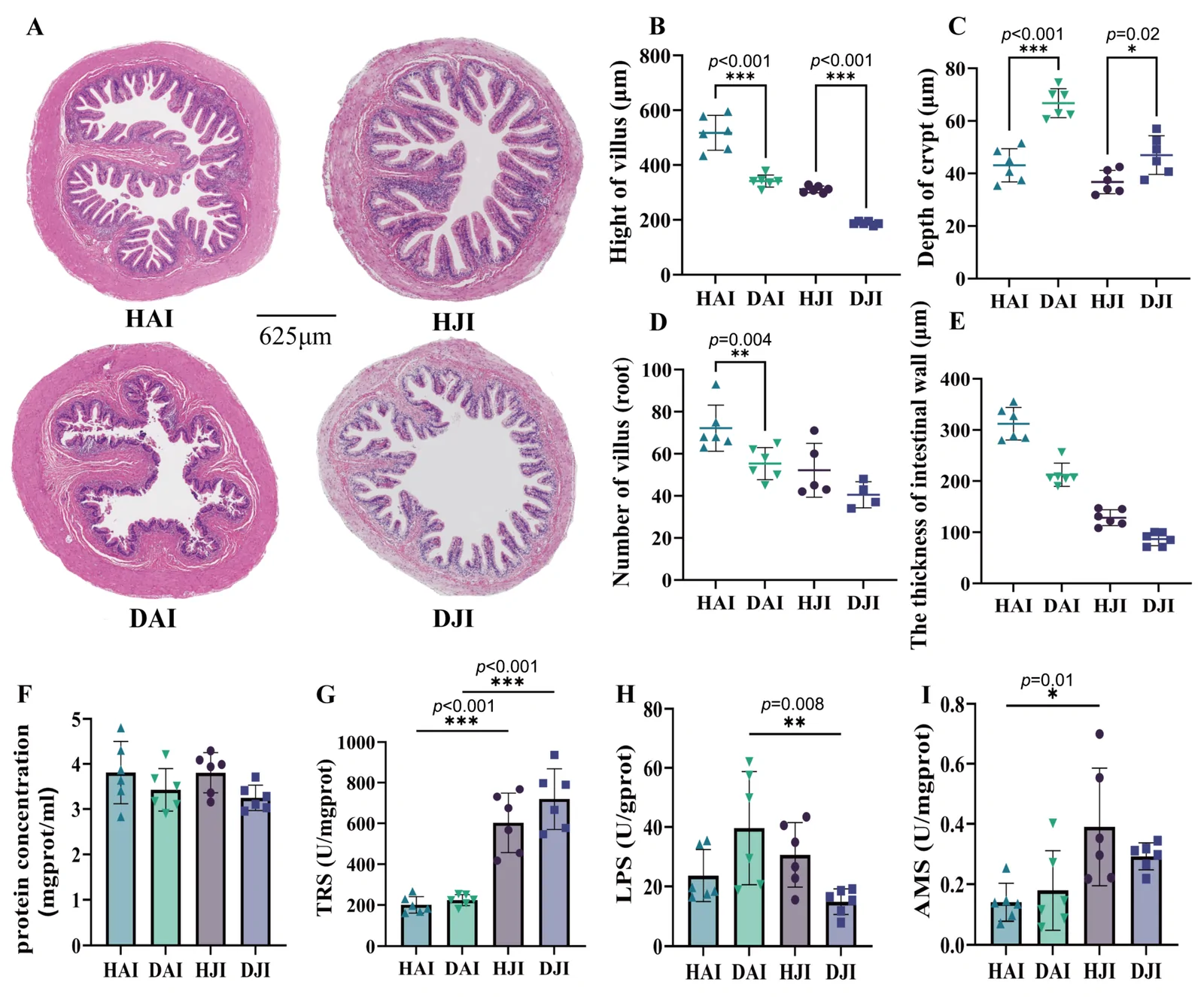
**The differences in intestinal morphology and digestive enzyme activity of turtles between different growth stages and physiological states (*n* = 5).** (**A**) Colon morphology (625 μm, ×20 full scans) of intestinal tissue sections of turtles. HAI, healthy adult turtle. DAI, diseased adult turtle. HJI, healthy juvenile turtle. DJI, diseased juvenile turtle. (**B**) Height of intestinal villi. (**C**) Depth of the intestinal crypt. (**D**) The number of intestinal villi. (**E**) The thickness of the intestinal wall. (**F**) The concentration of intestinal protein. The activities of digestive enzymes in the intestine. (**G**) TRS, trypsin. (**H**) LPS, lipase. (**I**) AMS, amylase. Two-way ANOVA; * *p* < 0.05, ** *p* < 0.01, and *** *p* < 0.001.

The morphological analysis of the colon revealed significant differences in villus length, crypt depth, and a higher number of villi of the intestinal tissue in turtles across different physiological states (Figure 1A). Both healthy juvenile and adult turtles exhibited a significantly longer villus length, many more villi, and a much thicker intestinal wall compared to their diseased counterparts (Figure 1B,D, *p* < 0.05). In contrast, the crypt depth was significantly lower in the healthy group than that of the diseased group (Figure 1C, *p* < 0.05).

In terms of digestive enzyme activity, no significant differences were observed in the content of intestinal protein or the activity of the three digestive enzymes between healthy and diseased turtles (*p* > 0.05). However, compared with adult turtles, juveniles exhibited significantly higher trypsin activity in both healthy and diseased groups (*p* < 0.001). In the healthy group, juvenile turtles showed significantly elevated amylase compared to adults (*p* = 0.01). Conversely, in the diseased group, juveniles displayed significantly lower lipase activity than adults (Figure 1G–I, *p* = 0.008).

### 3.2. Comparison of Antioxidant Enzyme Activity in the Liver

**Figure 2:**
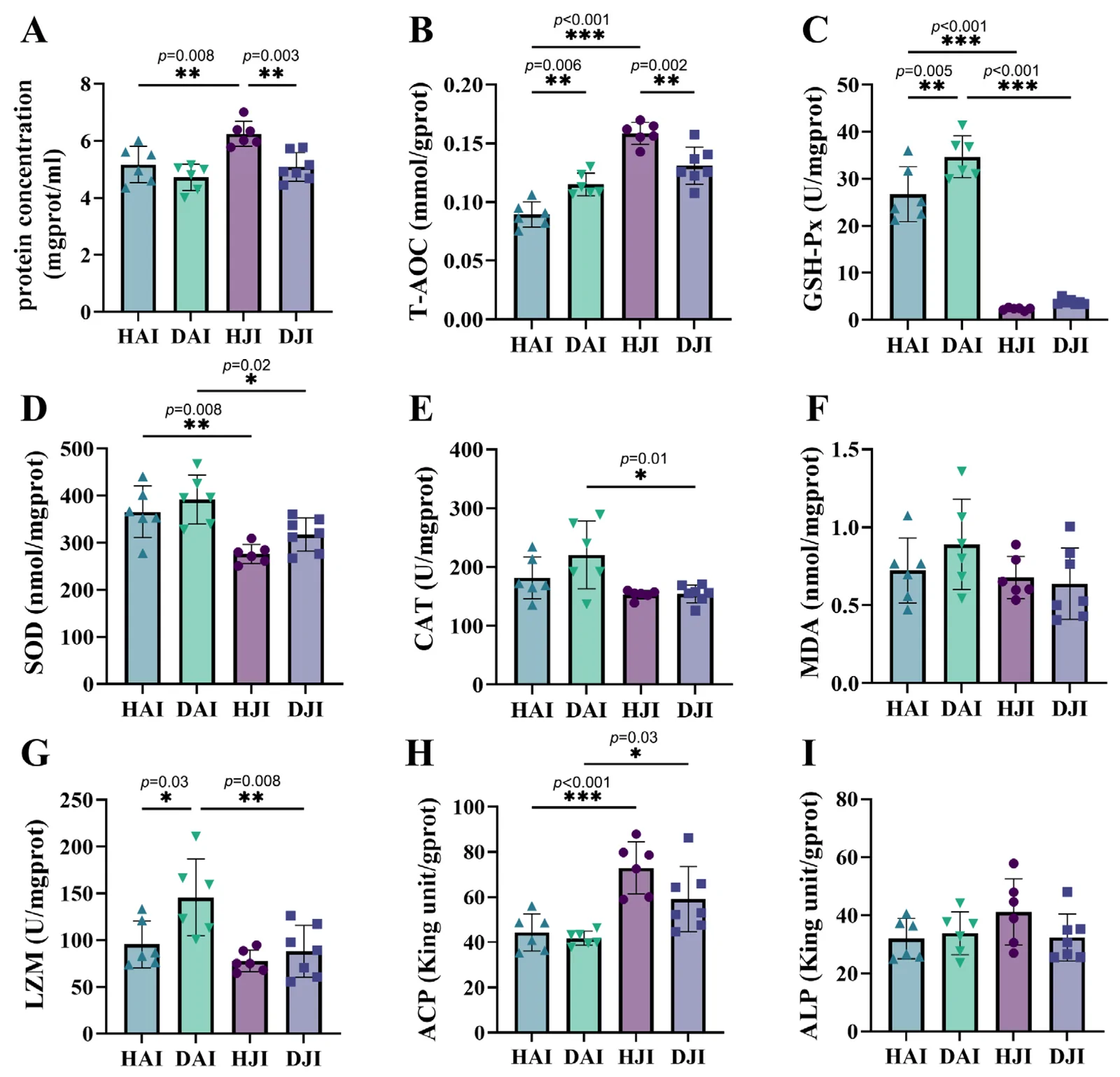
**Liver antioxidant capacity and non-specific immune-related enzyme activity of turtles at different growth stages and physiological states under greenhouse conditions (*n* = 5).** (**A**) Protein concentration of liver. (**B**) T-AOC, total antioxidant capacity. (**C**) GSH-Px, glutathione peroxidase. (**D**) SOD, superoxide dismutase. (**E**) CAT, catalase. (**F**) MDA, malondialdehyde. (**G**) LZM, lysozyme. (**H**) ACP, acid phosphatase. (**I**) ALP, alkaline phosphatase. Two-way ANOVA; * *p* < 0.05, ** *p* < 0.01, and *** *p* < 0.001.

The analysis of antioxidant enzyme activity in liver samples revealed notable differences based on physiological status. In the healthy group, adult turtles exhibited significantly higher activities of glutathione peroxidase (GSH-Px) and superoxide dismutase (SOD) compared to juvenile turtles (Figure 2C,D, *p* < 0.05). Conversely, the liver protein content, total antioxidant capacity (T-AOC), and alkaline phosphatase (ACP) enzyme activity were significantly lower in the adult group than in the juvenile group (Figure 2A,B,H, *p* < 0.05).

Under diseased conditions, the adult group demonstrated significantly higher activities of GSH-Px, SOD, catalase (CAT), and lysozyme (LZM) compared to the juvenile group (Figure 2C–E,G, *p* < 0.05), while acid phosphatase (ACP) activity exhibited an opposite trend (Figure 2H, *p* = 0.03). Additionally, the diseased adult group showed significantly elevated levels of T-AOC, GSH-Px, and LZM activity compared to the healthy adult group (Figure 2B,C,G, *p* < 0.05).

### 3.3. Comparison of Nutritional Composition in the Muscle

**Figure 3:**
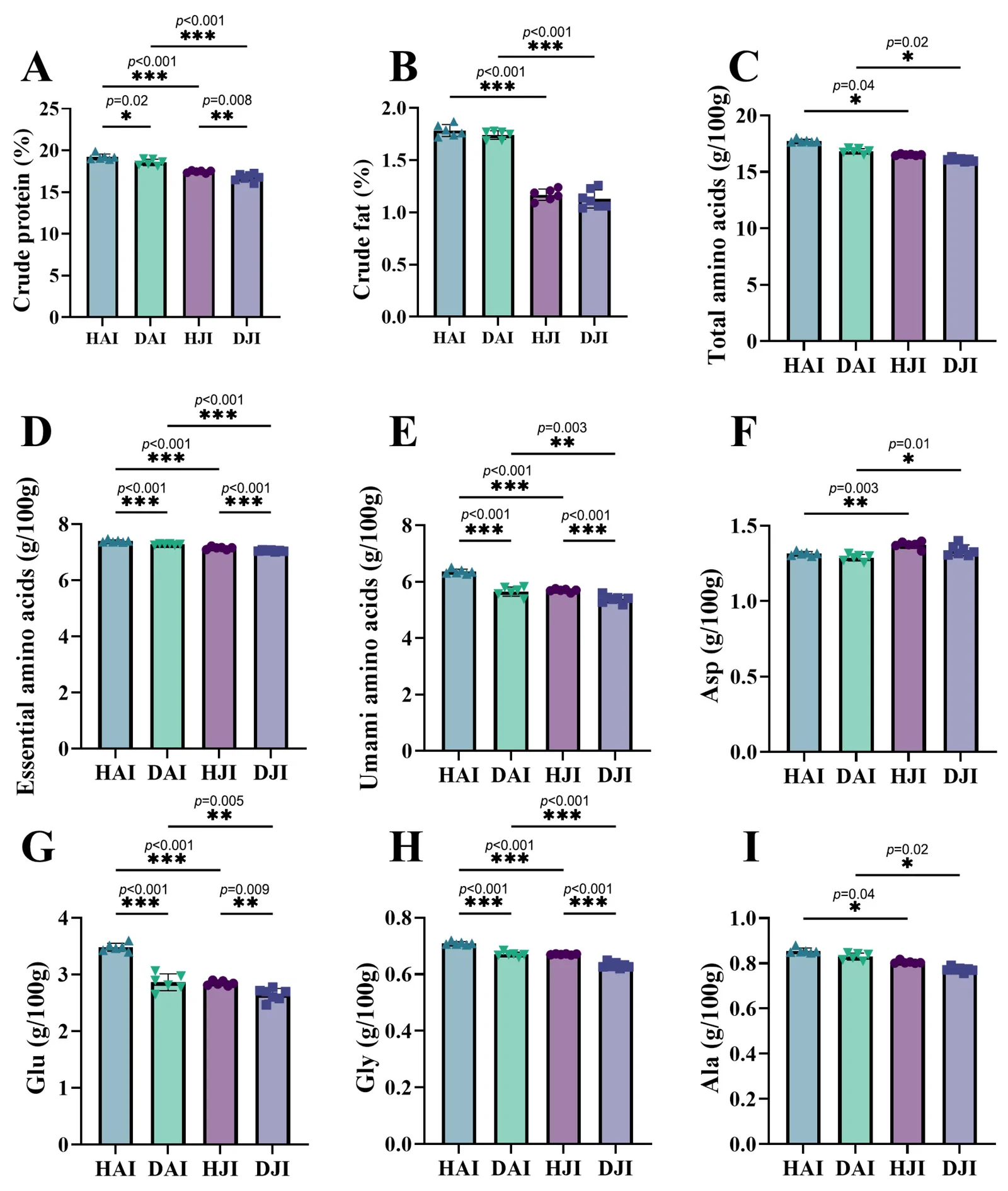
**Muscle nutritional composition of turtles at different growth stages and physiological states under greenhouse conditions (*n* = 5).** (**A**) Crude protein. (**B**) Crude fat. (**C**) Total amino acids. (**D**) Essential amino acids. (**E**) Umami amino acids. (**F**) Asp, aspartic acid. (**G**) Glu, glutamic acid. (**H**) Gly, glycine. (**I**) Ala, alanine. Two-way ANOVA; * *p* < 0.05, ** *p* < 0.01, and *** *p* < 0.001.

Analysis of the muscle samples indicated significant differences in nutritional content based on health status and growth stages. Under identical growth stages, the healthy adult group demonstrated markedly higher levels of crude protein, essential amino acids, umami amino acids, glutamic acid, and glycine compared to the diseased adult group (Figure 3A,D,E,G,H, *p* < 0.05). Similarly, the healthy juvenile group exhibited significantly elevated concentrations of these five indicators relative to the diseased juvenile group (Figure 3A,D,E,G,H, *p* < 0.05).

Moreover, when comparing groups within the same health status, the healthy adult group had significantly greater levels of crude protein, crude fat, total amino acids, essential amino acids, and four umami amino acids compared to the healthy juvenile group (Figure 3A–D, *p* < 0.05). Similarly, all measured nutritional parameters in the diseased adult group were significantly higher than those in the diseased juvenile group (Figure 3A–I, *p* < 0.05).

### 3.4. Comparison of Microbial Composition in the Intestine

**Figure 4:**
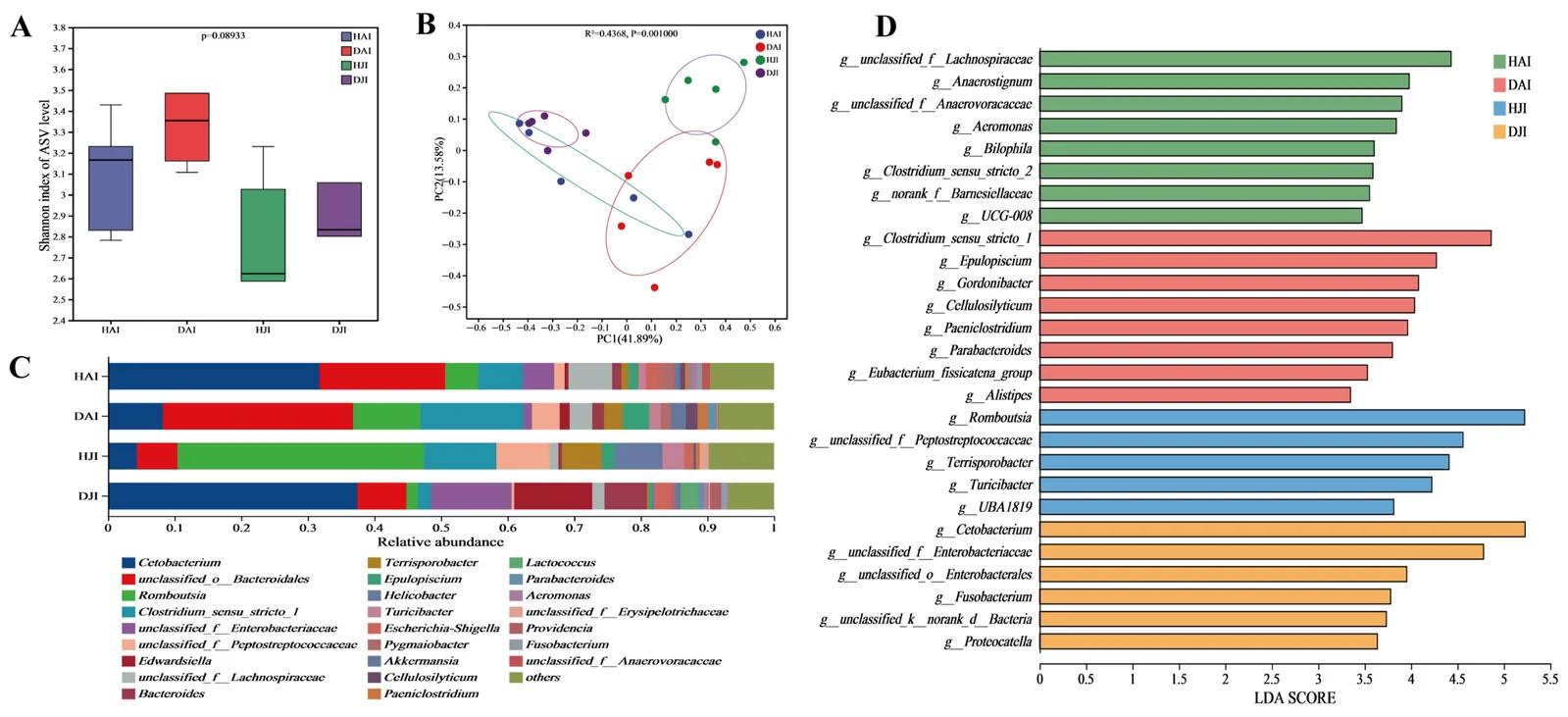
**Gut microbial diversity and composition of turtles at different growth stages and physiological states under greenhouse conditions (*n* = 5).** (**A**) Kruskal–Wallis H test for Shannon index. (**B**) The β-diversity of microbial compositions of various groups at the genus level. (**C**) The microbial composition of each group at the genus level. (**D**) The different microbiota between various groups based on LDA effect size (LEfSe) analysis.

The α-diversity of gut microbiota in turtles showed no significant differences between the two growth stages and two physiological states (Figure 4A, *p* > 0.05). In contrast, the microbial community composition of gut microbiota in turtles demonstrated significant differences among the four groups by principal coordinate analysis (PCoA) based on Bray–Curtis distances (*p* = 0.001, Figure 4B). Notably, the intestinal microbiota composition exhibited marked differences between adult and juvenile turtles, including between the healthy adult group and the healthy juvenile group (Group HAI/HJI, ANOSIM R^2^ = 0.4040, *p* = 0.004) (Figure S2B), and between the diseased adult group and the diseased juvenile group (Group DAI/DJI, ANOSIM R^2^ = 0.3877, *p* = 0.009) (Figure S2D). Moreover, significant differences also existed between healthy and diseased turtles. Specifically, the microbiota composition of the healthy juvenile group was markedly different from that of the diseased juvenile group (Group HJI/DJI, ANOSIM R^2^ = 0.4710, *p* = 0.004) (Figure S2C), but no significant differences were discovered between the healthy adult and diseased adult groups (Group HAI/DAI, ANOSIM R^2^ = 0.2346, *p* = 0.093) (Figure S2A).

At the genus level, the gut microbiota of the greenhouse turtles exhibited a predominance of *Cetobacterium*, followed by unclassified genera within the order of *Bacteroidales*, *Romboutsia*, and *Clostridium*_*sensu*_*stricto_1* (Figure 4D). Notably, *Cetobacterium* and five other genera were enriched in the diseased juvenile group. Eight genera, including *Clostridium*_*sensu*_*stricto_1* and *Epulopiscium*, were enriched in the diseased adult group. Additionally, *Romboutsia*, *Turicibacter*, and *Terrisporobacter* showed higher relative abundance in the healthy juvenile group, while *Anaerostignum* and *Aeromonas* demonstrated increased prevalence in the healthy adult group (Figure 4D).

### 3.5. Correlation Analysis of Gut Microbiota with Enzyme Activity and Muscle Nutritional Composition

**Figure 5:**
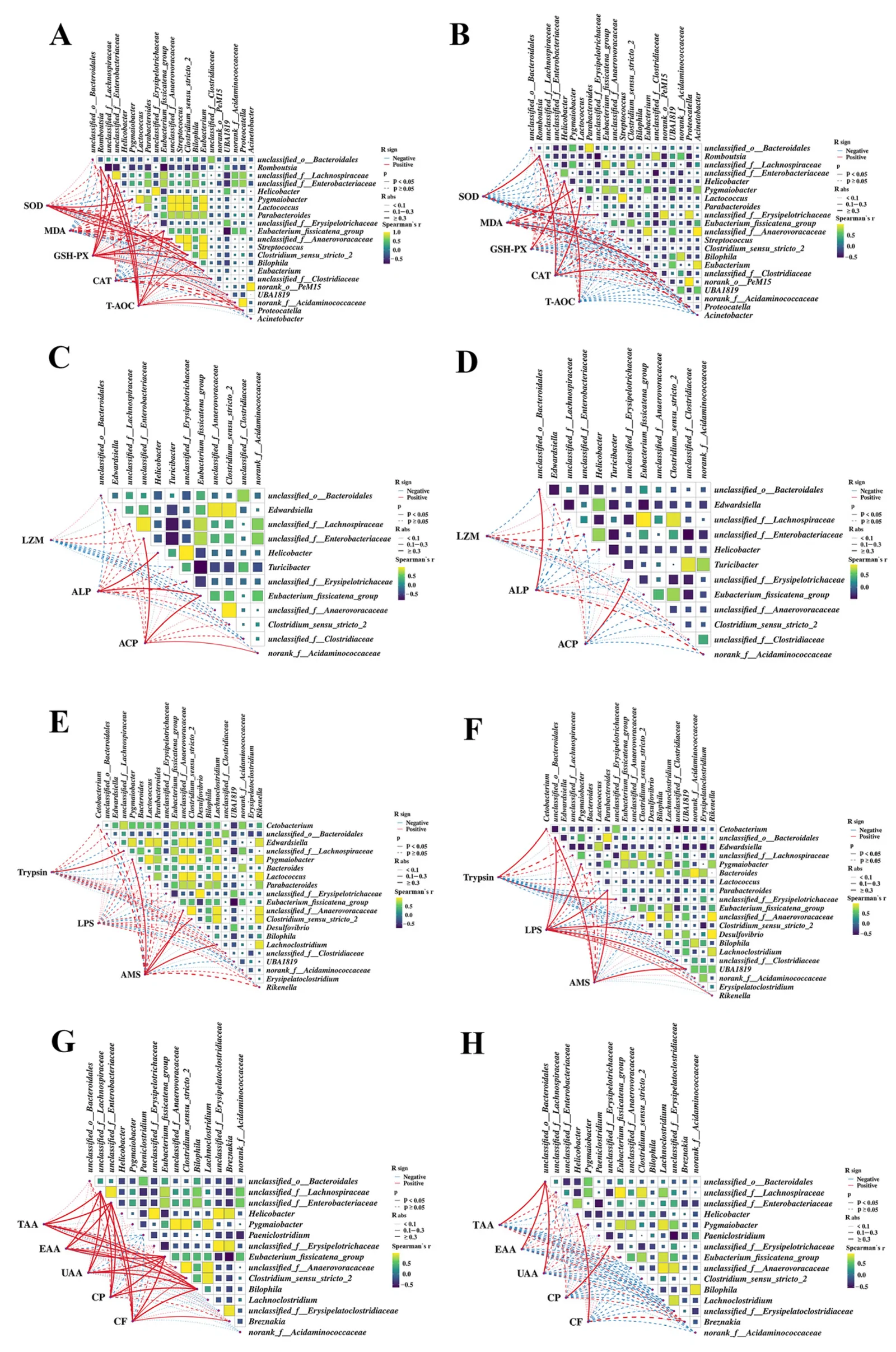
**Correlation network heatmap between gut microbiota at the genus level and indicators of antioxidant enzymes, immune enzymes, digestive enzymes, and muscle nutritional composition (*n* = 5).** Correlation between liver antioxidant enzyme activity and gut microbiota (**A**) in healthy Chinese soft-shelled turtles and (**B**) in diseased Chinese soft-shelled turtles. Correlation between liver non-specific immune enzyme activity and gut microbiota (**C**) in healthy Chinese soft-shelled turtles and (**D**) in diseased Chinese soft-shelled turtles. Correlation between intestinal digestive enzyme activity and gut microbiota in (**E**) healthy Chinese soft-shelled turtles and in (**F**) diseased Chinese soft-shelled turtles. Correlation between muscle nutrient content and gut microbiota in (**G**) healthy Chinese soft-shelled turtles and in (**H**) diseased Chinese soft-shelled turtles. Spearman’s rank correlation between bacterial genera with top 20% abundance. Mantel’s rank correlation between bacteria and each indicator.

Spearman correlation analysis identified numerous significant associations between the gut microbiota of the top 50% species (relative abundance above 0.1%) at the genus level and various physiological parameters, including liver antioxidant and non-specific immune enzyme activities, intestinal digestive enzyme activities, and muscle nutritional composition characteristics. In healthy Chinese soft-shelled turtles, liver antioxidant enzymes (GSH-Px) and intestinal digestive enzymes (AMS), along with muscle nutritional composition indicators including total amino acids, essential amino acids, umami amino acids, crude protein and crude fat, exhibited significant positive correlations with several bacterial taxa, such as *unclassified*_*f*_*Lachnospiraceae*, *unclassified*_*f_Anaerovoracaceae*, and *Clostridium*_*sensu*_*stricto*_*2* (Figure 5A,E,G). However, diseased individuals exhibited a significant reduction in the number of microbial genera positively correlated with key physiological parameters, including hepatic antioxidant enzymes, non-specific immune markers, and muscle nutritional components (Figure 5B,H). For example, GSH-Px activity only maintained a significant positive correlation with *Helicobacter* in diseased turtles (Figure 5B). The *unclassified*_*f_Erysipelotrichaceae* showed significant positive correlations with SOD, ACP, trypsin, and CP (Figure 5B,D,F,H). These results indicate that gut microbiota dysbiosis disrupts the host–microbiota–immune network, presenting different interaction relationships between gut microbiota and physiological indicators in healthy and diseased states. 

### 3.6. Predictive Analysis of Microbial Function of Different Groups

**Figure 6:**
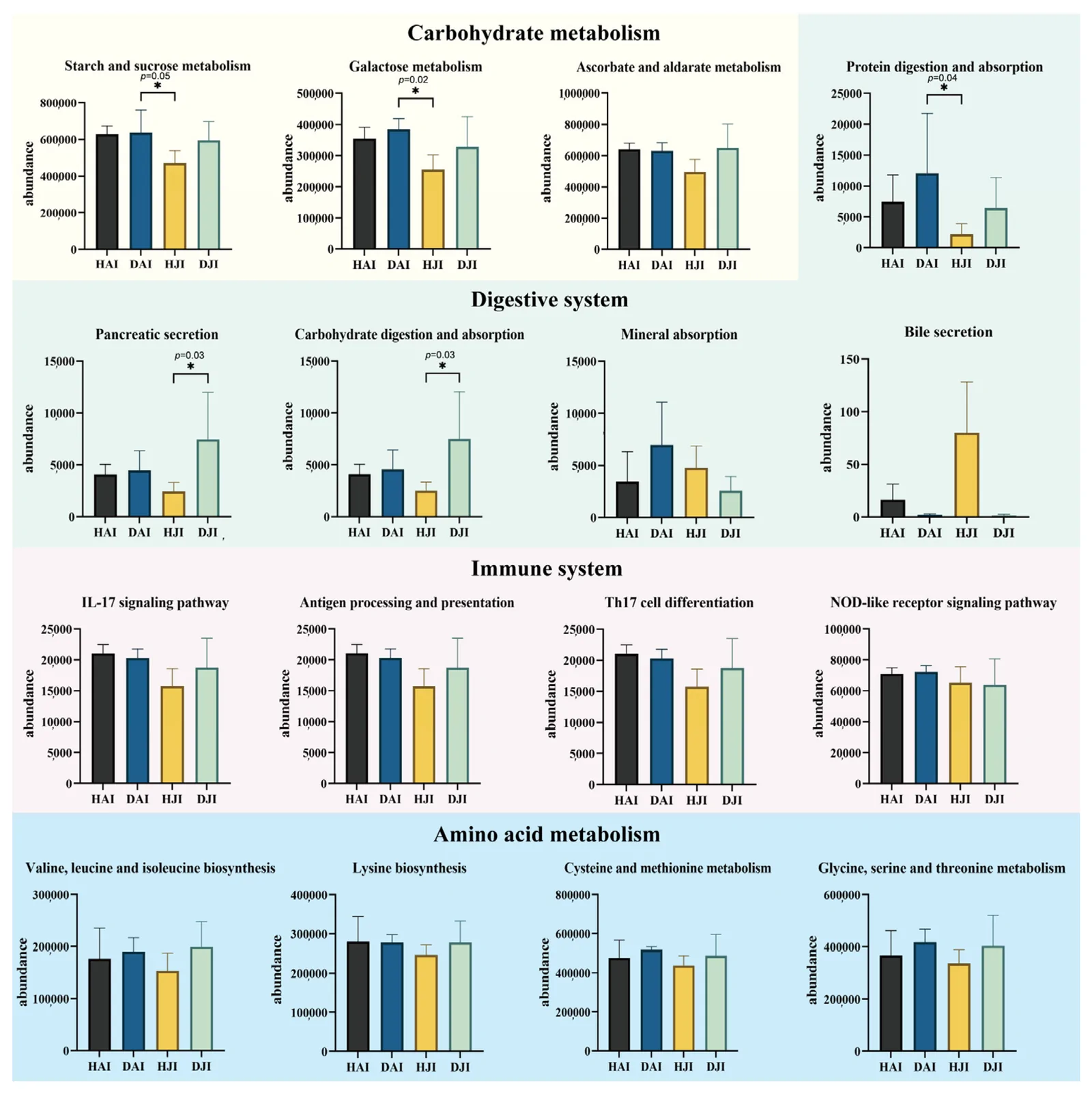
**Differences in the functional metabolism pathways of gut microbiota among turtle groups at different growth stages and physiological states under greenhouse conditions (*n* = 5).** Different background colors represent different KEGG metabolic pathways at Level 2, * *p* < 0.05.

Based on the functional prediction analysis through PICRUSt2, this study preliminarily elucidated the functional characteristics of the gut microbial community in soft-shelled turtles. The results reveal that in the carbohydrate metabolic pathways, the functional abundances of galactose metabolism and starch and sucrose metabolism in diseased adult turtles were significantly higher than those in healthy juvenile turtles. As for digestive system functions, diseased adult turtles exhibited higher functional abundances in protein digestion and absorption compared to healthy juveniles (Figure 6, *p* < 0.05). Notably, diseased juvenile turtles showed significantly higher functional abundances in pancreatic secretion and carbohydrate digestion and absorption than their healthy counterparts (Figure 6, *p* < 0.05). In contrast, no significant differences were observed in functional immune system or amino acid metabolism pathways among the four groups. However, the abundance of functional pathways of these two types in the HJI group was relatively lower (Figure 6, *p* > 0.05).

### 3.7. Correlation Analysis of Gut Microbiota and Its Functional Metabolic Pathways

**Figure 7:**
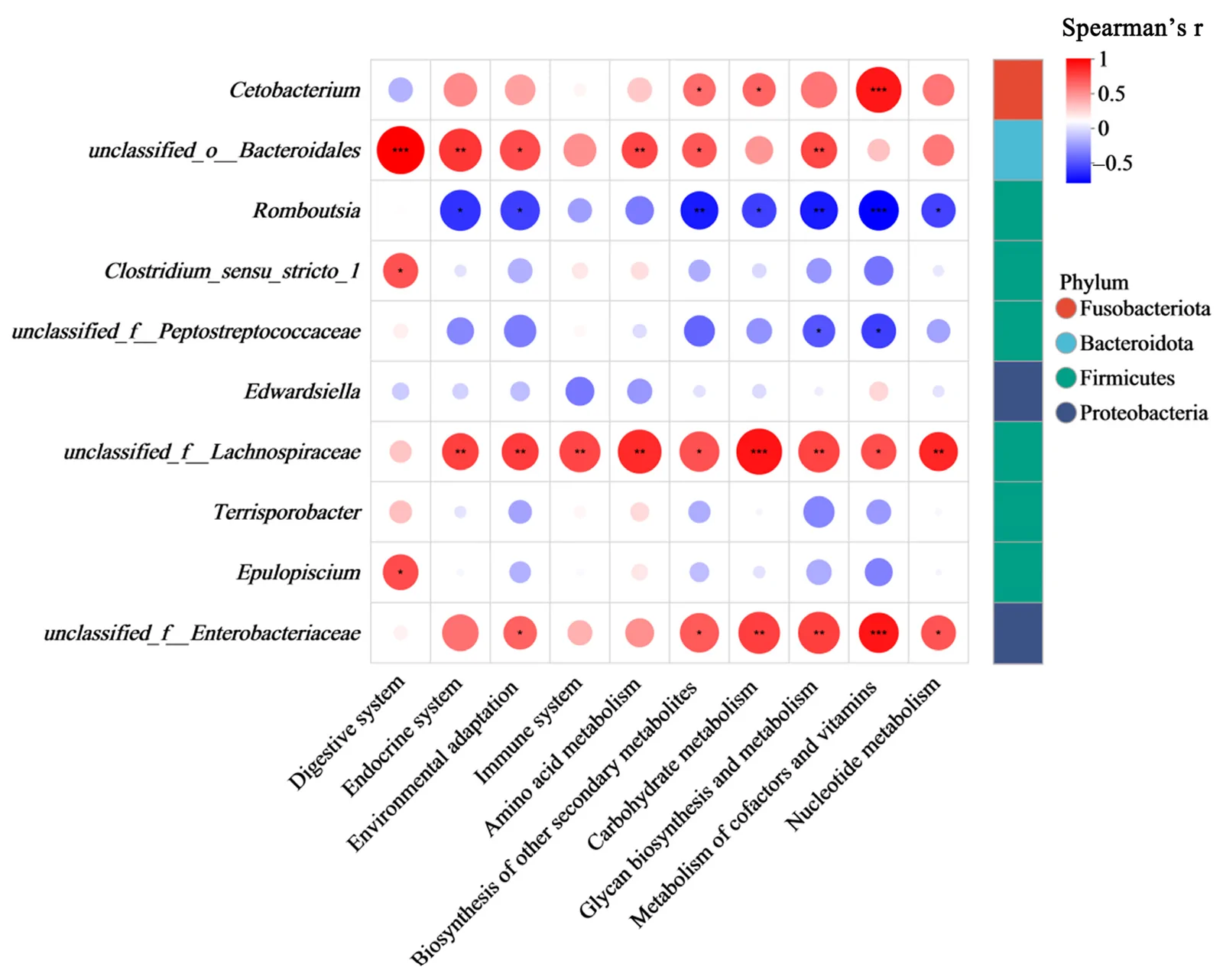
**Correlation analysis of gut microbiota and its functional metabolism pathways (*n* = 5).** Spearman’s rank correlation test: * *p* < 0.05, ** *p* < 0.01, and *** *p* < 0.001.

Spearman correlation analysis was carried out between the functional abundance of KEGG secondary pathways and the top 10 genera of the gut microbial community. It was found that *unclassified_o_Bacteroidales*, *unclassified_f_Lachnospiraceae*, and *unclassified_f_Enterobacteriaceae* were significantly positively correlated with metabolism pathways, such as environmental adaptation, biosynthesis of other secondary metabolites, and glycan biosynthesis and metabolism. *Romboutsia* and *unclassified_f_Peptostreptococcaceae* were significantly negatively correlated with functional pathways of glycan biosynthesis and metabolism of cofactors and vitamins. Among them, *unclassified_f_Lachnospiraceae* was significantly positively correlated with the immune system, and *unclassified_o_ Bacteroidales* and *unclassified_f_Lachnospiracea* were significantly positively correlated with amino acid metabolism (Figure 7).

## 4. Discussion

### 4.1. Intestinal Morphological and Digestive Enzyme Activity

Intestinal villi constitute essential morphological features for nutrient absorption in the small intestine [31,32]. In our study, healthy turtles exhibited significantly longer villi compared to diseased individuals. Yuan et al. have also reported that intestinal damage could impaire the growth of microvilli [33]. In addition, intestinal crypts serve as primary loci for epithelial secretion and regeneration [34]. However, deeper crypts may signify inflammation, heightened immune responses, or developmental changes. For instance, bacterial infections can induce gut inflammation and lead to an increase in the depth of the crypt [35]. Our results also found deeper crypts in the gut of diseased turtles, suggesting that disease may lead to crypt elongation. Furthermore, the thickness of the intestinal mucosa is closely linked to nutrient absorption and transport efficiency. Yuan et al. found that the damaged intestinal wall became thinner and began to dissolve, accompanied by mucosal congestion in *Cyprinus carpio* var. *jian* [33]. We found that diseased turtles exhibited a significantly thinner posterior intestinal wall compared to their healthy counterparts. Meanwhile, congestion and dark red discoloration occurred in the intestines of diseased individuals during dissection. Therefore, based on the observed pathological evidence, we infer that the intestines of the diseased turtles likely exhibit structural or functional impairment.

The digestive and absorptive functions of the intestine mainly depend on the activity of intestinal digestive enzymes, the majority of which are secreted by the pancreas prior to entering the small intestine [36]. For example, trypsin is widely present in animals and plays a crucial role in enhancing protein digestion [37]. Yuan et al. found that the activities of protease, lipase, and amylase in the proximal intestine were significantly higher in healthy tissues compared to diseased ones of *Jian carp* [33]. Similarly, our study showed that the lipase and amylase activities of healthy juvenile turtles were significantly higher than those of diseased juvenile turtles. Moreover, the intestines of these healthy juveniles displayed better morphological integrity. All the results suggest that the disease might lead to intestinal damage, accompanied by a decline in digestive capacity. Furthermore, Kuz’mina examined the effect of age on digestive enzyme activities in several freshwater teleost species, and found that total enzyme activity increased with age under certain conditions [38]. In our study, adult turtles exhibited significantly higher lipase activity than juvenile turtles with WAD. However, this trend did not translate to healthy turtles, and might be influenced by the host’s genetics, the breeding environment, or even the sample size. Taken together, these observations suggest that digestive enzyme activities varied with both developmental stage and health status, reflecting a dynamic adaptation of the digestive system to physiological changes.

### 4.2. Liver Antioxidant and Non-Specific Immune Enzyme Activity

A clear developmental trajectory was observed regarding antioxidant defense. Adult turtles possessed significantly enhanced activities of crucial antioxidant enzymes (GSH-Px, SOD) compared to juveniles. GSH-Px is a crucial enzyme for decomposing peroxides, while SOD serves as a key antioxidant defense enzyme, which can convert highly reactive superoxide anions into hydrogen peroxide [39]. These findings suggest that the maturation of the antioxidant system enhances oxidative stress resilience in adulthood [40]. Furthermore, we also observed that the T-AOC, GSH-Px, and LZM activities in the livers of diseased adult turtles were significantly higher than those of healthy adults, which indicates that disease heightens oxidative stress. For example, the prolonged cadmium exposure of crucian carp triggered oxidative stress, disrupted the antioxidant balance, and elevated oxidative markers in the liver and kidneys [41]. Lysozyme, as a widely distributed hydrolase, plays a crucial role in the biological defense system by degrading the cell walls of pathogenic microorganisms [42]. Siwicki et al. have also reported that the serum lysozyme activity of *Cyprinus carpio* increased during the early stages when infected with *Pseudomonas alcaligenes* and *Aeromonas punctata* [43]. In addition, ACP serves as a biomarker for evaluating the health and immune responses of aquatic organisms [44]. An earlier study has revealed that healthy greater amberjack (*Seriola dumerili*) exhibit higher ACP activity, while diseased individuals show a marked decline with heat stress [45]. The result indicates that disease might inhibit enzyme activity or disrupt its metabolic pathways [46]. Our findings also show that ACP levels were significantly lower in diseased turtles than those of their healthy counterparts. Moreover, juvenile turtles displayed notably higher ACP levels compared to adults. Safi et al. also found that protease and ACP activities increased during the early growth stages of cuttlefish [47]. Hence, evaluation of the immune capabilities of aquatic species requires the incorporation of various biochemical parameters.

### 4.3. Nutritional Composition in the Muscle

Many studies have indicated that substantial differences exist in muscle composition and nutritional content between juvenile and adult aquatic animals [36]. For example, adult *Larimichthys crocea* have been reported to contain higher levels of collagen and inosine monophosphate in their muscle tissues compared to juveniles [48,49]. Our results demonstrate significant differences in the nutritional composition of muscle tissues between juvenile and adult turtles. Specifically, adults exhibited substantially higher concentrations of crude protein, total amino acids, essential amino acids (EAAs), and umami amino acids. Notably, the elevated levels of key EAAs like lysine, leucine, and glutamic acid in adult muscle were critical for both protein synthesis and healthy development. For example, higher protein and EAA levels were found to be important nutritional components in the growth of juvenile slider turtles [50]. It was also observed that Chinese soft-shelled turtles have better growth performance when fed with brown fishmeal containing higher levels of EAAs [51]. Similarly, the levels of these muscle nutritional indicators were also significantly higher in healthy turtles than in diseased turtles. Previous studies have revealed that disease can alter metabolism, reduce muscle protein content, and be associated with changes in overall nutritional value in Chlamys nobilis, captive mammals, and reptiles [52,53]. In summary, adult turtles possess a nutritionally richer muscle profile, particularly in protein and essential amino acids, while disease significantly compromises muscle quality and nutritional value.

### 4.4. Gut Microbiota Composition and Its Relationship with Digestion, Immunity, and Meat Quality

Gut microbiota are widely recognized to be closely associated with host health and metabolic processes [54,55]. Previous research on aquatic animals and turtles has reported distinct gut microbiota diversity and composition between healthy and diseased individuals, as well as across different growth stages [56,57]. In the current study, within the same growth stage, the Shannon diversity index was notably higher in the diseased group compared to the healthy group. Such inconsistent trends across studies are likely attributable to multiple confounding factors shaping gut microbiota. We speculate that WAD is linked to disrupted gut microbiota homeostasis and the overgrowth of potential pathogenic taxa, because the gut microbiota of diseased aquatic animals tends to be more susceptible to fluctuations in rearing water microbiota [58]. Li et al. also demonstrated that gut microbiota between juvenile and adult *Rimicaris kairei* was significantly different regarding the evenness and richness of bacterial species [59]. Meanwhile, investigations on the developmental stages of bighead carp (*Hypophthalmichthys nobilis*) have revealed that shifts in gut microbial communities are associated with variations in muscle free amino acid and fatty acid profiles [60]. Consistent with these reports, our study detected obvious differences in gut microbial community structure between juvenile and adult soft-shelled turtles.

Furthermore, gut microbiota are closely associated with host immune status. Disturbances in the gut microbiome have been linked to altered immune responses and increased susceptibility to pathogen infection in diseased aquatic animals [61]. In addition, antioxidant supplementation is correlated with shifts in gut microbiota composition and function, which may relieve stress responses in hosts [62]. Multiple studies have noted that probiotics are associated with enhanced immune performance in aquatic animals alongside balanced gut microbiota and reduced disease risk [63]. For example, *Romboutsia* are known to influence the levels of host antioxidant enzymes like GSH-Px and SOD [64]. Levels of alkaline phosphatase (ALP) are related to chronic inflammation and have been shown to be associated with the abundance of *Turicibacter* [65,66]. In our findings, *Turicibacter* exhibited significant positive correlations with ALP activity. Collectively, these observations reflect complex associations among gut microbiota, immune responses, and host metabolic status in aquatic animals.

Gut microbiota are also associated with host nutrient utilization via correlations with the activity and expression of digestive enzymes [67,68,69]. Many studies have indicated that certain probiotics are associated with higher activity of key digestive enzymes (e.g., amylase, lipase, and protease) and improved digestive efficiency in hosts. [70,71]. For example, decreased abundance of *Cetobacterium* has been linked to lower serum lipopolysaccharide (LPS) concentrations [72]. In our study, *Cetobacterium* was enriched predominantly in diseased juvenile turtles and displayed significant negative correlations with lipase activity. This finding coincides with the lowest lipase activity observed in the DJI group (Figure 1H). Previous studies have also indicated that aquatic gut microbiota are associated with the biosynthesis of diverse nutrients in hosts. For example, gut microbial communities are correlated with free amino acid and fatty acid levels in bighead carp (*Aristichthys nobilis*), further linked to variations in muscle nutritional composition [60]. Alterations in amino acid profiles have also been associated with specific bacterial taxa, such as *unclassified_f_Lachnospiraceae* [73,74]. In line with these results, *unclassified_f_Lachnospiraceae* was enriched in the gut of healthy adult turtles (HAI group) in our research. This taxon showed significantly positive correlations with multiple nutritional indices (TAA, EAA, UAA, CP, and CF), all of which were elevated in the HAI group. These correlations imply that *unclassified_f_Lachnospiraceae* may be associated with variations in muscle nutritional composition; however, such correlational relationships were altered under diseased conditions (Figure 5G,H).

Regarding predicted gut microbial functions, elevated predicted capacity for protein digestion and absorption in adult turtles, especially diseased individuals, may reflect an adaptive trait to meet elevated protein demand during growth or disease. The abundance of *unclassified_f_Lachnospiraceae* was significantly positively correlated with amino acid metabolism pathways predicted by PICRUSt2. It should be noted that these metabolic profiles represent inferred functional potential rather than directly measured microbial activity, implying potential microbiota-linked compensatory mechanisms facilitating nutrient uptake under pathological conditions [75]. Collectively, these results reveal complex correlational networks linking gut microbiota with host digestion, immune status, and muscle nutritional composition, which partly helps to interpret the complicated pathological processes occurring in Chinese soft-shelled turtles.

### 4.5. Limitations and Future Research Directions

White abdominal disease severely restricts the sustainable development of Chinese soft-shelled turtle aquaculture owing to its high transmissibility, long incubation period, and high mortality. Its complex etiology—frequently involving polymicrobial infections, particularly by *Aeromonas hydrophila*—and severe pathological manifestations, including hepatopancreatic necrosis and intestinal atrophy, contribute to its detrimental impact [76]. Although antibiotic use has partially mitigated disease outbreaks, their overuse has led to escalating concerns regarding antimicrobial resistance, residual contamination, and environmental hazards, underscoring the urgent need for eco-friendly and sustainable alternatives [77]. In this context, microecological agents such as probiotics have garnered considerable attention due to their favorable safety profile, absence of residue accumulation, lack of contribution to antimicrobial resistance, and multifaceted mechanisms of action—including modulation of gut microbiota and enhancement of host immune responses. Evidence indicates that supplementation with *Bacillus subtilis* contributes to hepatic health and improves metabolic function in *P. sinensis* [78], while chitosan oligosaccharides and β-glucan have been shown to bolster antioxidant capacity and immunological performance through regulation of intestinal microbial composition [79]. These findings suggest that integrating microecological interventions with precision management of rearing conditions—such as water quality and thermal regulation—may enable the establishment of a comprehensive health management system effective in mitigating WAD outbreaks. Nevertheless, the mechanistic interplay between intestinal homeostasis and disease susceptibility in Chinese soft-shelled turtles remains insufficiently understood, representing a critical knowledge gap that must be addressed to facilitate the targeted development of high-efficacy microecology.

This study identified close correlations among gut microbiota, immune enzyme activity, and muscle nutritional composition in *P. sinensis*. Nevertheless, several limitations should be acknowledged. First, the experiment was carried out on one population of greenhouse-cultured *P. sinensis* from a single farming region. Regional variations in water quality, feed, and stocking density were not taken into account, so the universality of our findings needs further verification under diverse farming conditions. Second, our research only performed comparative and correlation analyses of physiological indices, partial muscle nutrients, and gut microbiota between healthy and diseased juvenile and adult turtles. Multi-omics approaches including metagenomics and metabolomics should be adopted in follow-up work. Future research aims to resolve the functions of core microbial taxa, isolate and verify key functional strains, and uncover the molecular pathways whereby gut microbiota modulate host physiological traits. These explorations will facilitate the development of targeted probiotic products for healthy turtle farming.

In addition, each group had five turtles (*n* = 5), with every individual serving as an independent biological replicate. The relatively small sample size may reduce statistical power when analyzing numerous indicators simultaneously, potentially increasing the chance of false-positive or false-negative results. Larger sample sizes are required in subsequent studies to validate our conclusions.

## 5. Conclusions

Under controlled greenhouse conditions, this study comprehensively demonstrated the differences in hepatic antioxidant status, immune function, digestive capacity, gut microbiota, and muscle nutritional components between juvenile and adult *P. sinensis*, as well as between healthy individuals and individuals infected with WAD. Healthy turtles exhibited superior colonic morphology, characterized by increased villus density, thicker intestinal walls, and higher digestive enzyme activities. Additionally, adult turtles showed stronger liver antioxidant capacity and better muscle nutritional composition, while similar results were observed in healthy individuals compared to diseased ones. Notably, gut microbiota composition correlated significantly with host immunity, digestive capacity, and muscle nutritional profiles, with microbial interaction networks showing marked differences between healthy and diseased states. Collectively, these observations may provide preliminary references for optimizing the production performance of greenhouse-cultured *P. sinensis*. Future research should expand the scope of regions, strains, and farming conditions to verify the generalizability of the results, integrating multi-omics technologies to decipher key microbial functions and molecular mechanisms (e.g., functional genes, metabolite pathways) and isolate functional strains for validation, thereby offering targeted strategies for the development of probiotics for the healthy farming of *P. sinensis*.

## Data Availability

All 16S rRNA gene sequencing data were uploaded to the NCBI SRA database with the accession number PRJNA1188359 (accessed on 20 November 2024).

## Supplementary Materials

The following supporting information can be downloaded at: https://www.mdpi.com/article/10.3390/antiox15091100/s1, Figure S1: Flow chart of experimental design; Figure S2: Principal coordinate analysis (PCoA) of the microbial composition of the gut of turtles based on weighted UniFrac distances; Table S1: Diet and nutritional composition of juvenile and adult turtles.
