# Supplementary material for: Effects of Growth Stage and Physiological State on Gut Health, Antioxidant Capacity, and Muscle Nutritional Composition in Greenhouse-Cultured Pelodiscus sinensis
Source: Antioxidants (Basel). 2026 Aug 31;15(9):1100. doi: 10.3390/antiox15091100 (PMC13603482; doi:10.3390/antiox15091100)

Supplementary material

Table S1. Diet and nutritional composition of juvenile and adult turtles.

Diet composition	Juvenile turtles	Adult turtles
White fish meal	43.20%	35.50%
Alpha-amylase (pre-gelatinized)	20.50%	24.40%
Fermented soybean meal	17.00%	20.00%
Beef liver powder	4.50%	5.50%
Brewer's yeast powder	3.40%	4.40%
Corn gluten meal	3.40%	4.40%
Corn oil	2.80%	2.20%
Soybean enzymatic hydrolyzed protein	2.30%	1.70%
Calcium dihydrogen phosphate	1.10%	0.90%
Choline chloride	0.30%	0.30%
Composite vitamin premix	0.20%	0.20%
Composite mineral premix	0.20%	0.20%
Appetizer	0.90%	0.20%
**Total**	**100%**	**100%**

Nutritional composition	Juvenile turtles	Adult turtles
Crude protein	47.60%	43.70%
Crude fat	3.80%	3.20%
Crude fiber	1.80%	2.50%
Crude ash	18.50%	21.80%
Calcium	6.20%	4.50%
Total phosphorus	3.50%	2.60%
Lysine	3.45%	4.60%
Methionine	0.95%	1.30%
sodium chloride	4.70%	5.90%
Moisture	9.50%	9.90%
**Total**	**100%**	**100%**

Figure S1. Flow chart of experimental design.

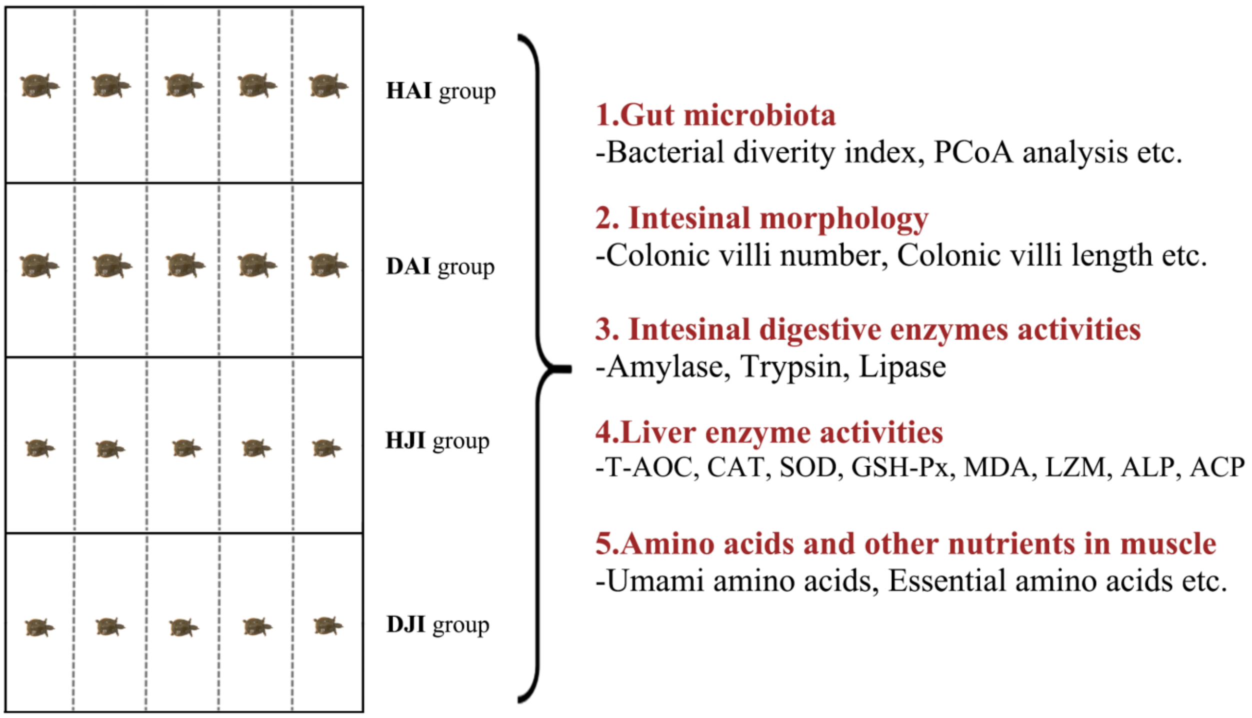

Figure S2. Principal coordinate analysis (PCoA) of the microbial composition of the gut of turtles based on weighted UniFrac distances. The difference of microbial community structure (A) between healthy adult turtles (HAI) and diseased adult turtles (DAI) in greenhouse farming conditions. (B) between healthy adult turtles (HAI) and healthy juvenile turtles (HJI) in greenhouse farming conditions. (C) between healthy juvenile turtles (HJI) and diseased juvenile turtles (DJI) in greenhouse farming conditions. (D)between diseased adult turtles (DAI) and diseased juvenile turtles (DJI) in greenhouse farming conditions

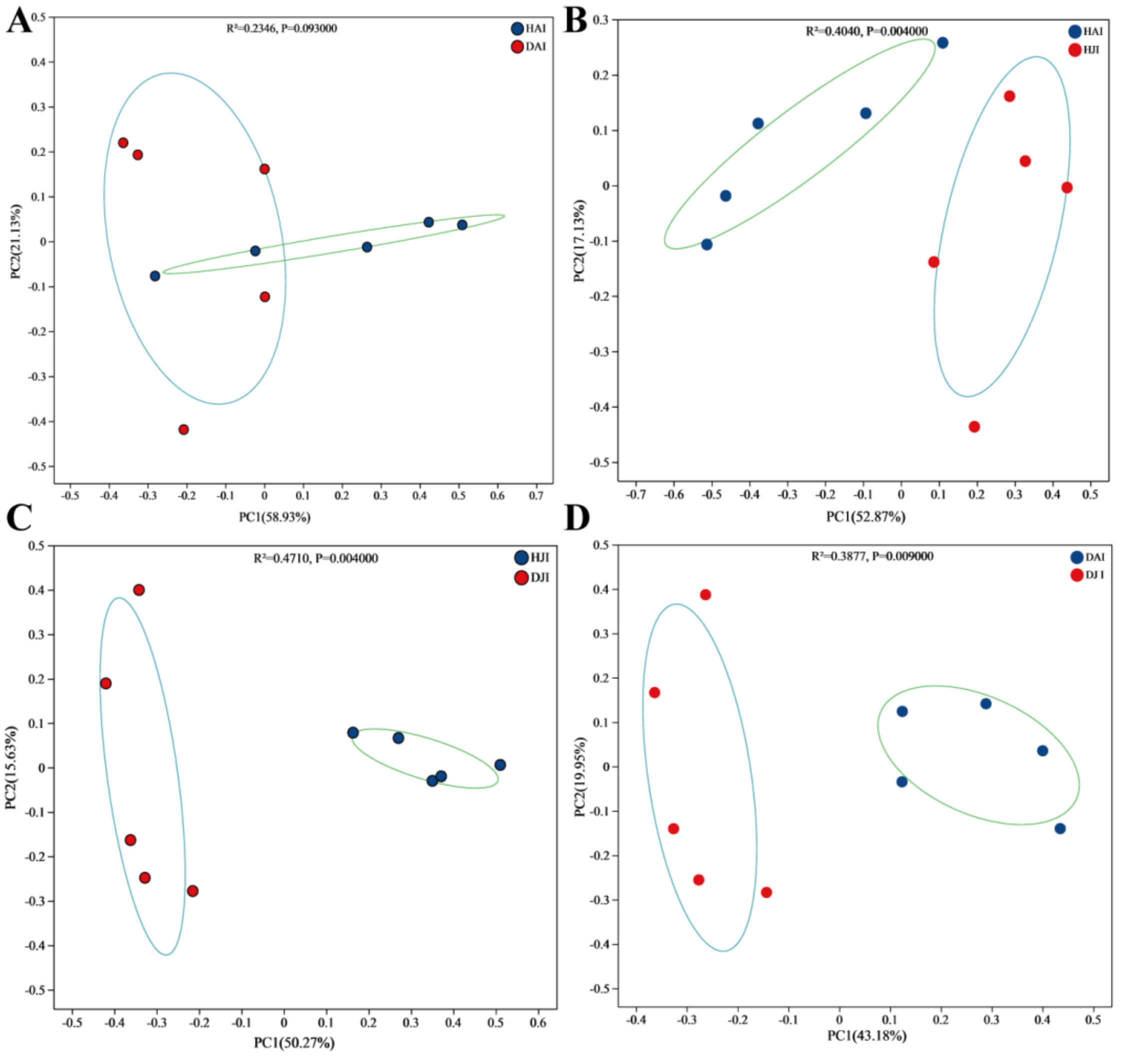

Supplement: Supplementary file 1 [file antioxidants-15-01100-s001.zip › antioxidants-4487787-supplementary.pdf]
